# 

**Published:** 1897-11-20

**Authors:** 


### EARLY EDITION.

**Notices of Births, Deaths, and Marriages** are inserted in this column at a charge of 2s. 6d. for an announcement not exceeding four lines.

### NOTICE TO CORRESPONDENTS.

All MS., Letters, Books for Review, and other matters intended for the Editor should be addressed The Editor, "The Hospital," The Hospital Building, 28 & 29, Southampton Street, Strand, W.C.

The Editor cannot undertake to return rejected MS., even when accompanied by stamped directed envelope.

**Notice to Advertisers and Subscribers.**

All Advertisements, Orders for Copies of The Hospital, and Business Communications should be addressed to **THE MANAGER, (Not to The Editor),** The Hospital, The Hospital Building, 28 & 29, Southampton Street, Strand, London, W.C.

The Cost of Subscription, post free, is as follows:—Per week, 2½d. per quarter, 2s. 8d.; per half-year, 5s. 3d.; per annum, 10s. 6d. Foreign:—Per annum, 15s. 2d.; payable, in all cases, in advance.

**NOTICE.—Vol. XXII. of "The Hospital" (April, 1897, to September, 1897), in handsome cloth binding, is now on sale at the Office of this Journal, price 7s. 6d. Cases for binding the Numbers contained in this Volume, 1s. 6d. Index to Vol. XXII., 2½d., post free.**

**The Monthly Part for December will be ready on December 1st, Price 6d., by post 8d.**

### BOOKS RECEIVED.

T. Fisher Unwin.

"Masters of Medicine: William Harvey." By D'Arcy Power, F.S.A., F.R.C.S.

Charles Griffin and Co.

"Manual of Nursing, Medical and Surgical." By Lawrence Humphrey, M.A., M.D. (Sixteenth Edition.)

Spottiswoode and Co.

"Sick-room Cookery." By Maude Earle. With Notes on the Feeding of Infants, by Frank C. Madden, F.R.C.S.

"Western Mail," Cardiff.

"Handbook to the Workmen's Compensation Act, 1897." By M. Roberts-Jones. (Third Edition.)

Kegan Paul, Trench, and Co.

"The Elements of Hypnotism." By Ralph Henry Vincent,

S. W. Partridge and Co.

"The Zenana." Vol. IV.

William Blackwood and Sons.

"An Indian Romance."

Baillière, Tindall, and Cox.

"Air, Food, and Excercises." By A. Rabagliati, M.A., M.D., F.R.C.S.

Scientific Press, 28 & 29, Southampton Street. Strand.

"Burdett's Official Nursing Directory, 1898." By Sir Henry Burdett, K.C.B.

**Periodicals and Pamphlets Received.**—The Queenslander, The Anglo-Russian, Medical Press, Leeds Hospital Magazine, Guyoscope, Literary Digest, Educational News, London, Windsor Magazine, Nursing Notes, Knowledge.

### SCRAPS AND GLEANINGS.

The work on the new convalescent home generously presented to the Derbyshire Royal Infirmary by Mr. Herbert Strutt has been started. The new building will cost about £5,000, and in addition Mr. Strutt has started an endowment fund with £3,000.

The medical staff at the Leicester Infirmary are agitating for an increase in the resident medical and surgical staff. In the course of a discussion on the subject at a recent meeting, it was stated that the work which the hospital had to do was getting out of all proportion to the ability of the institution to deal with it. The funds of the infirmary were not equal to its requirements, and the town would have to face very shortly the necessity of according the institution still further support.

